# Equity Measurement of Public Sports Space in Central Urban Areas Based on Residential Scale Data

**DOI:** 10.3390/ijerph19053104

**Published:** 2022-03-06

**Authors:** Ying Liu, Huan Wang, Cheng Sun, Huifang Wu

**Affiliations:** School of Architecture, Harbin Institute of Technology, Harbin 150006, China; wanghuan@hit.edu.cn (H.W.); suncheng@hit.edu.cn (C.S.); wuhuifang@hit.edu.cn (H.W.)

**Keywords:** public sports facilities, spatial equity, network analysis, multi-source data, traffic mode

## Abstract

Urban public sports facilities have captured much public attention because of their close ties to public health. However, few studies have comprehensively assessed the equity of accessibility to various types of public sports space with a fine scale. This study proposed a spatial equity measurement method based on multi-source urban data and GIS network analysis. Residential buildings were taken as the minimum research unit to investigate the equity differences of residents’ enjoyment of urban public sports space accessible by walking and public transportation. Taking Harbin, China, as an example, this study calculated and visualized the proximity of more than 12,000 residential buildings to a variety of public sports space in the central urban area. The results showed that: (1) urban centers enjoy more public sports space resources than border areas, that is, the developed area has more advantages than the emerging area; (2) according to the classification of sports space, their spatial distribution pattern and measurement results are obviously different; (3) the areas with a low walking equity degree also had a low bus equity degree. This study integrated multi-source data into the traditional spatial computing models and provided an important reference for the equitable planning of urban public sports space.

## 1. Introduction

As China’s urbanization and industrialization accelerate, urban residents do not merely enjoy a higher standard of living, but also face more and more health problems, the most important of which are chronic diseases, weight gain and obesity. According to the Report on Nutrition and Chronic Diseases in China (2020) released by the National Health Commission of the People’s Republic of China, deaths caused by chronic diseases accounted for 88.5% of the total deaths in 2019, with more than half of the adult population suffering overweight or obesity and unhealthy lifestyles still prevalent [1]. According to the World Health Organization, active physical activity can remarkably benefit the health of urban residents and prevent and control non-communicable diseases including chronic diseases such as cardiovascular disease, cancer, and diabetes which are often caused by obesity [2]. As the basic media of sports, public sports space is undoubtedly important to residents’ health. A number of studies have shown that proximity to sports space is associated with an increase in physical activities and has a positive impact on health [3,4,5,6].

Sports space includes places where people can perform physical exercise, such as parks and squares where leisure activities are the main forms of exercise, fitness centers that focus on strength and shape building, and all kinds of stadiums or facilities where more intense aerobic exercise can be conducted. According to the 2014 National Fitness Report issued by the General Administration of Sport of China, the most popular public sports space for people over the age of 20 includes public stadiums, fitness paths, squares, parks and fitness clubs [7]. The planning and layout of public sports spaces in China generally follows the principles of “fairness, efficiency, convenience, centrality, combination of concentration and decentralization, and layout in combination with residential space structure”. The allocation of park green space will be set according to its classification and service population, service radius and per capita indicators. The standard of urban land classification and planning construction land in China (GB50137-2011) also determines that the minimum per capita park green space area is 8 square meters per person. Even so, Harbin only raised the per capita park green space index from 7.5 square meters (2014) to 10 square meters in 2020 and many other cities are still working towards this goal. In addition, as a spatial entity, sports space is influenced by economic, political, and social factors, so it may not be equally enjoyed by all people. Existing studies have pointed out that there is obvious spatial inequity in the distribution of many types of facilities [8,9,10]. At the same time, the results of studies may be different [6]. At present, public sports space is the main place for residents to exercise, but problems remain in the contradiction between the increasing sports demand of the masses and the scarcity of sports facilities and playing field resources.

Spatial equity refers to the degree of equal distribution of services or amenities in different regions and economic, ethnic, and political groups [11]. Its assessment index can be summarized as spatial accessibility and availability, that is, the proximity of people to space and the amount of space available. Many studies have focused on the equity of sports space, mainly on a certain type such as parks [10,11] and sports facilities [8]. Few studies have analyzed the equity of various types of sports space [6,12]. At the same time, there are often differences in the focus and calculation methods adopted in similar studies, which are the key reasons for different results, mainly in the following three aspects.

### 1.1. Literature Review

#### 1.1.1. Computational Models of Spatial Equity

One of the most common and relatively simple approaches is the container approach, which measures the accessibility by determining whether there is space to examine within a particular geographic cluster [6], such as a street, a neighborhood, or a census district. Of course, it can also take a specific walking distance as the range of the container and calculate the value of equity through the amount, area, or percentage of the space examined within the area [13], which is similar to buffer analysis. The container approach is simple but is also open to criticism. First, it only computes the objects within the container, so there are serious boundary problems, especially when based on typical geographic clustering units. At the same time, the analysis results based on different size units may be different. Secondly, the space homogenization of container can be a problem. The container approach assumes that people in containers enjoy equal space equity, which is obviously unrealistic. Especially when the size of containers is too large, the conclusion may lead to serious ecological fallacies.

Kernel density estimation can be regarded as an improved method based on the container approach. It calculates the decreasing value from the target space to the critical distance through the kernel function and the bandwidth, and then fits the result into a smooth cone surface. The advantage of kernel density estimation lies in that it can assign values to all study areas [6] and bring distance attenuation into a fair range of investigation, thus overcoming the spatial homogenization caused by the container approach [12]. However, the biggest problem in kernel density estimation lies in the choice of bandwidth, because bandwidth is the most critical factor to determine the analysis results, and the results calculated by different bandwidth are significantly different. Bandwidth represents the similarity and correlation between kernel density estimation and the container approach.

The improvement of the above two methods is mainly reflected in the introduction of distance as a variable and its influence on the results and the consideration of spatial heterogeneity. Another approach is also based on distance, such as proximity analysis and travel cost approach. They assess the equity of the population to the target space by calculating the cost distance, and the distance is in an inverse relation to the equity degree. However, the results of simple computational logic, such as measuring the distance between a house and the nearest park or calculating the average distance to all facilities within a certain range, are unconvincing [14]. Similarly, the widely applied gravity model incorporates more non-spatial factors, such as facility size and attraction, on the basis of distance attenuation [12]. The distance calculation method has also experienced the transformation from Euclid distance and Manhattan distance to network distance based on the urban road network. With the development of network analysis module of GIS, more and more vector-based road network distance assessment methods have been applied to the study of spatial equity [10,15,16].

#### 1.1.2. Basic Research Unit

The application of the container approach has also aroused attention to the smallest unit of spatial analysis. Since it often uses the smallest integrated unit of data available, such as zip code, community, or census area. In addition to the inconsistency of the analysis results caused by these different unit choices, the general characteristics of the units can not reflect internal differences, and the range of units is still too large for individuals. The larger the unit, the more likely it is to ignore spatial heterogeneity, idealize the results, and draw conclusions that deviate from reality [11,12]. This is due to the limitations of the data itself, and many studies have encountered this problem. However, people are very interested in decisions that are relevant to their fields and directly influence them. As the spatial scale moves from local to regional and eventually national, fewer and fewer people are interested in these issues [17].

It is worth noting that the smallest analysis unit used in previous studies is a single building [11,18], such as a residential building, which can be closer to the real situation of individuals, and the integration results based on this larger area are more reasonable. At the same time, the analysis from a single building also unifies the research scale, and there will not be differences due to the change in countries or regions. However, there is little research on a building as the basic analysis unit, mainly because there are few channels to acquire such data. The advent of detailed, open and accessible multi-source urban data, such as road network data, point of interest (POI) data and building data, has made it easier to conduct studies at a finer scale, providing insight into aspects that were previously difficult to assess [19].

#### 1.1.3. Estimation of Travel Capacity

In this paper, travel capacity refers to the maximum range of people’s activities in a certain mode of transportation under the limitation of a certain standard (such as time), which represents the accessibility of the crowd to the target space. For example, the container approach or buffer analysis applies a specific walking distance as the maximum range of units [11,20,21], and targets outside the range are not included in the calculation results. However, there is no unified standard for the setting of people’s walking ability or walking distance, 400 m, 500 m, 800 m, 1000 m or even more are adopted, which also makes people question the research. However, many theories or studies have little difference in defining travel times. For example, the new urbanist definition of walkability includes most things within a 10-min walk away from home [22]. In the United States Park Scoring Index, a 10-min walk from home is also used as the assessment standard for urban park access [23]. In China’s 2018 Urban Residential Planning and Design Standard, 15-min, 10-min and 5-min life circles were used as step control scales of residential areas [24], and living space was organized based on walking distance. Shanghai, Jinan, and other cities have also launched corresponding planning of 15-min walking living circles. Shanghai has also introduced a 30-min sports life circle, requiring access to a sports venue within 30 min. Moreover, in China, the concept of “life circle” refers to the allocation of various functions and facilities required by residents’ basic life based on the walking range within a certain period of time. The walking distance of 10–15 min is equivalent to 800–1000 m, and many studies also use this time or distance as the research basis [10,15,16,25].

Taking time as the standard can unify the research scale and avoid large differences caused by different countries or regions. However, walking as the only mode of travel remains open to problems. The most direct is that walking distance can only be defined as a range. In addition to the boundary effect, it cannot fully represent the travel behavior and ability of the crowd and cannot reflect the real spatial equity situation. Some studies suggest that accessibility should be highly sensitive to modes of travel [26]. There are also more studies that consider multiple modes of transportation to assess spatial equity [27,28,29]. Integrating various modes of transportation can be closer to the real travel situation of people and have a more real assessment of spatial equity.

### 1.2. Research Content

The research problem of this paper is how to effectively measure and evaluate the equity of public sports space in central urban areas. Our objectives include: (1) to provide a simple and detailed method for equity assessment of public sports space in the city; (2) to provide reference for the planning and configuration optimization of public sports space.

The structure of this paper is as follows: the second part is a detailed introduction of research data sources and usage methods. The third part is to carry out the practice and provide the analysis and results of measurement. The fourth part summarizes the main contributions of this study and discusses its application to planning practice. The last part summarizes the results of the survey, which can provide a basis for the policy and practice of sports spatial planning.

## 2. Materials and Methods

In order to provide a more effective and applicable spatial equity measurement method and evaluate the urban public sports spatial equity as accurately as possible, this paper takes Harbin city as an example and proposes a comprehensive assessment method of public sports space in central urban area. Our research is based on the concept of lifetime circle, using multi-source urban data and GIS network analysis, taking residential buildings as the basic analysis unit, and combining walking and public transportation modes.

### 2.1. Study Area

**Figure 1:**
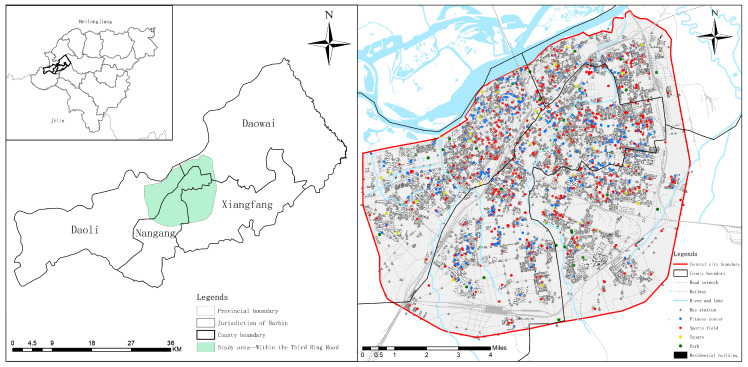
Study area and data examples.

This case study area is the central urban area of Harbin, mainly including 4 districts within the Third Ring Road (Figure 1). The 4 urban districts have a permanent population of 4.09 million, with a population density of about 2711 people/km^2^. Harbin, located in the northernmost part of China, is a big city with the highest latitude and lowest temperature nationwide. Harbin has four distinct seasons. Winter is long and cold, summer is short and cool, and the temperature in spring and autumn drastically changes and the time is short. The unique geographical location shapes the prosperous ice and snow culture and ice sports in Harbin, but also makes the daily physical exercise greatly influenced by the climate. Walking and public transportation are equally important. The highway traffic of Harbin city is developed, showing the pattern of “one axis, four rings and eleven lines”. The rail traffic construction is still in development, and there are three subway lines. At present, most of the research on the equity of accessibility to sports space in China is concentrated in the south and first-tier cities, but few in cities in cold regions. At the same time, Harbin is also carrying out the 15-min and 30-min fitness circle plan, and the study of urban public sports space equity boasts practical significance and long-term value for the inspection of the existing scheme and future urban planning.

### 2.2. Data Preparation

Our research is to measure the urban spatial situation, so we need to obtain multi-source data, including basic geographic information, statistical data, and some emerging big data. This research focuses on the public sports space in central urban areas, i.e., urban sports space open to the public, both paid and free. Some sports spaces open only to a limited number of people are excluded, such as school stadiums which are not open to the public. According to the 2014 National Fitness Report and previous sports space classification [8,18,30], combined with the actual situation of Harbin, this article will divide public sports space into four categories, namely, parks and squares for leisure sports, fitness centers for strength training and body shaping, and all kinds of sports venues and facilities for intense aerobic exercise. The data of parks, squares, fitness centers, sports facilities and bus stations required by the study sourced from the POI data of Amap open platform (https://lbs.amap.com/, accessed on 10 September 2020). The data of residential building were obtained from Baidu Map and vectorized (https://lbsyun.baidu.com/, accessed on 27 November 2020); the information on the urban road network was obtained from OpenStreetMap (https://www.openstreetmap.org/, accessed on 29 November 2020). The final data included 12,548 residential buildings, 1159 sports fields, 1686 bus stops and 7661 roads (Figure 1 and Table 1).

The size and grade of sports space often represent the number of services it can provide. For example, the size of park has been regarded as a key factor influencing the accessibility of parks in previous studies [10,13,29]. Therefore, in order to standardize the analysis, we divided parks and squares into 5 grades (i.e., 5, 4, 3, 2 and 1) according to their area and fitness centers and sports facilities according to their service levels to represent their service capacity. In addition, other features of sports space are also important factors influencing people’s access, such as the number of facilities, quality of service and space experience, etc. [4,6], but these factors are difficult to quantify. Dianping (http://www.dianping.com/, accessed on 10 March 2021) is China’s leading platform of local information and trading, where people can freely post comments and scores on the places they visit, and the system will generate comprehensive scores of public facilities based on these scores. Facility score can represent the comprehensive evaluation of facilities by the public to a certain extent. We obtained the scores of four types of sports space from Dianping as the comprehensive assessment value of the people for the space (Table 1).

### 2.3. Study Design and Calculation Methods

#### 2.3.1. Study Design

**Figure 2:**
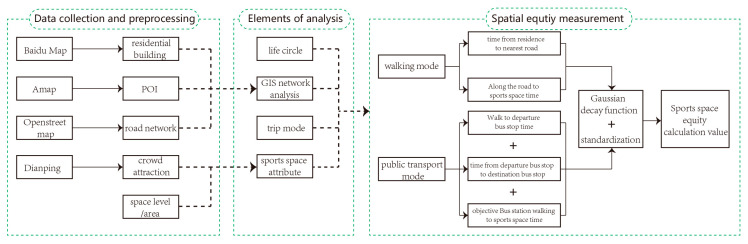
Conceptual and analytical framework of public sports space equity measurement.

After obtaining the required data, we need to import it into ArcGIS software for pre-processing, convert it into spatial data, eliminate invalid information and establish a database. The main method of research measurement is related to the sports space that can be achieved under certain circumstances starting from residential buildings, including the attributes of the sports space itself. The tool adopted in the research is the network analysis module in ArcGIS, and the key control information required for the module operation includes time/distance, speed/mode of transportation, corresponding to 15 and 30 min in the concept of “life circle”. At the same time, the mode of transportation also requires attention. Compared with single mode, spatial equity analysis under multi-mode travel can provide more realistic assessment [29] because it takes diverse consideration of travelling ability from the human scale. Therefore, the measurement of public sports space equity in central urban areas in this study includes 2 parts: the sum of calculated values of sports space accessible from residential buildings within 15min (about 1000 m) by walking and within 30min by bus [28]. In this part, we use ArcGIS network analysis module to work out the shortest path from residential buildings to sports space within two-time circles. The overall calculation process is shown in Figure 2 [19,28,30].

#### 2.3.2. Computational Method of Accessibility Equity to Sports Space by Walking

In the concept of 15-min walking circle, we assume that people can walk to the public sports space which is about 1000 m away from home (human walking speed is calculated at 1.2 m/s). Total travel time includes the time from home to the nearest road plus the minimum time along the road to the public sports space. We can calculate the spatial equity in the walking mode in the following formula [19,29]:

(1) $$A_{j}=\underset{i\in \left(t_{ij}\leq t_{o}\right)}{\sum }S_{i}\times P_{i}\times G\left(T_{ij}\right)$$

(2) $$G\left(T_{ij}\right)=e^{-\frac{1}{2}{\left(\frac{t_{ij}}{t_{0}}\right)}^{2}}$$

where $A_{j}$ indicates the equity value of public sports space from residential building j in walking mode; $t_{ij}$ represents the shortest walking time from residential building j to sports space i; $t_{0}$ is the maximum time by walking, i.e., 15 min; $S_{i}$ shows the service capacity of sports space i, namely, the level of sports space classified above; $P_{i}$ represents the comprehensive assessment value of sports space i by the people, namely, the score of Dianping; $G\left(T_{ij}\right)$ indicates a simplified Gaussian time decay function, which can represent the change in the influence of the walking time on spatial equity. It should be pointed out that after all of the calculations, we standardized the results according to the categories of sports space, that is, we assumed that the four types of sports space were equally important.

#### 2.3.3. Computational Method of Accessibility Equity to Sports Space by Public Transportation

In the concepts of 15-min walking circle and 30-min travel circle, we assume that people can travel by public transportation to the sports space which can be reached within 15-30min. The calculation of bus travel time includes three parts: the walking time from home to departure bus station ($w_{j}$), from departure bus station to destination bus station ($p_{ij}$) and the walking time from destination bus station to sports space ($w_{i}$). We calculate the spatial equity in the mode of public transportation in the following formula [19,28,30]:

(3) $$A_{j}=\underset{i\in \left(t_{ij}\leq t_{o}\right)}{\sum }S_{i}\times P_{i}\times G\left(T_{ij}\right)\times N_{i}\times N_{j}$$

(4) $$T_{ij}=w_{i}+p_{ij}+w_{j}$$

where $A_{j}$ is the equity value of public sports space from residential building *j* in the mode of public transportation; $t_{ij}$ is the shortest bus travel time from residential building j to sports space i; $S_{i}$ and $P_{i}$ have the same meaning as in formula (2); The calculation method of $G\left(T_{ij}\right)$ is the same as formula (2); $t_{0}$ is the longest time by bus, i.e., 30 min; $N_{i}$ is the number of bus stations within 400 m of sports space i; $w_{i}$ is the average time to walk to all bus stations within the range; $N_{j}$ is the number of bus stops within 600 m around residential building j; $w_{j}$ is the average time to walk to all bus stops within the range; $p_{ij}$ is the travel time from the departure bus station to the destination bus station. According to the data of Harbin Transportation Bureau, the bus travel speed is calculated at 10 km/h. Similarly, after all of the results were calculated, we standardized the results.

## 3. Results

### 3.1. Spatial Distribution Results

**Figure 3:**
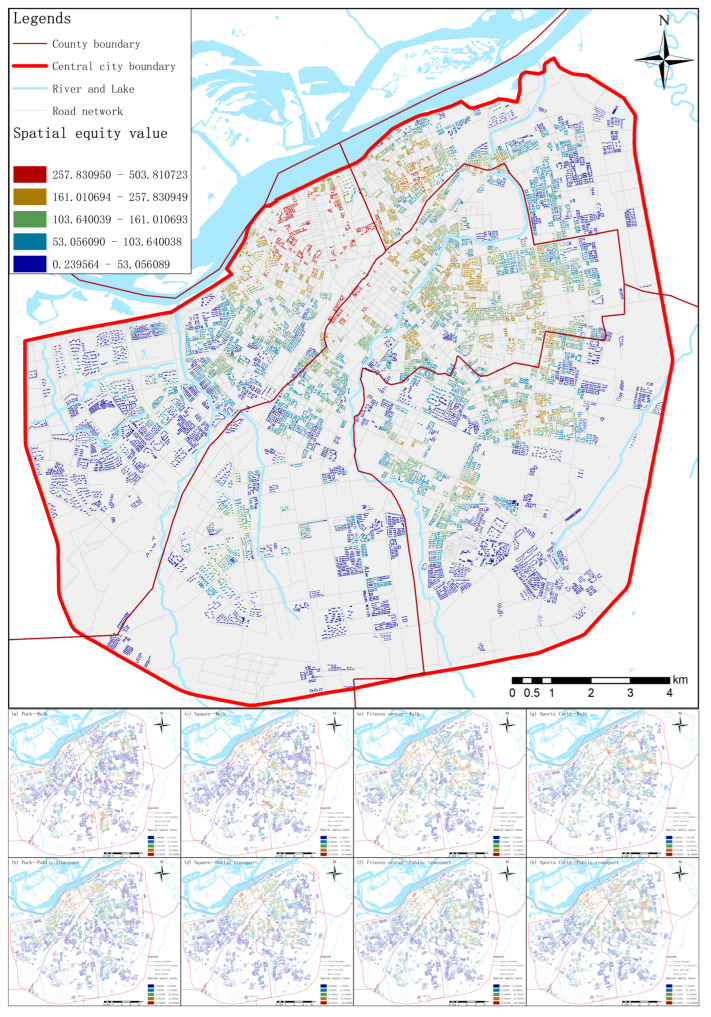
Equity distribution map of sports space in central urban area.

Figure 3 shows the visual status of sports space equity in the central urban area as a whole and in different traffic situations. On the whole, there is obvious an inequality of sports space in the inner city which shows a trend of gradual attenuation from the central area to the periphery. The margin of the four districts and the large area in the south of the city are all areas with low spatial equity values. At the junction of Daoli, Daowai and Nangang districts, the spatial equity value is relatively high. Meanwhile, this area is also the city center, with developed transportation and concentrated resources. In addition, the overall equity layout is not a single outward attenuation, the internal changes in the four districts are uneven. Different streets, and even different residential areas, will have noticeable differences in spatial equity.

The results of different transportation modes each showed the inequity of sports space, especially parks and squares. Apart from the walking pattern to the park, other spatial equity results also showed a trend of weakening from the city center to the periphery, and the spatial location of the overall distribution was similar to the comprehensive calculation results. Compared with other types of sports space, the change in equity classification of parks is not moderate, and low-value areas account for a high proportion of the total. The high-value regions of all kinds of cases are mainly concentrated at the junction of the three districts, and their distribution ranges are almost the same with minor differences. At the same time, due to the influence of sports space, traffic stations and urban road network distribution, there are discontinuous spatial equity changes in different modes. Compared with the comprehensive calculation results, the calculation results under classification change more obviously, and the spatial transition is not smooth.

### 3.2. Numerical Statistical Results

**Figure 4:**
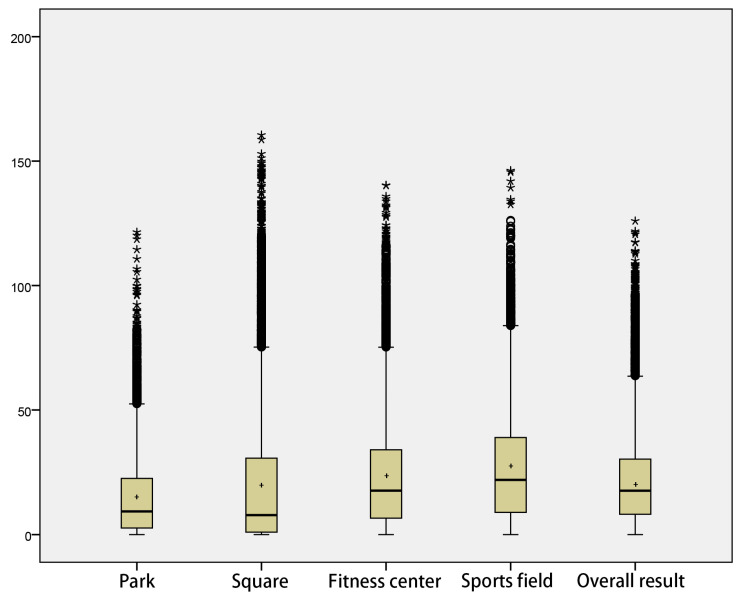
Box diagram of sports space equity in central city. The asterisk represents the outliers and the plus represents the average of the calculated data.

The spatial visualization results let us know which specific regions in the city have unequal spatial distribution of sports, and we also need to compare the degree of differences of such inequity. As shown in Table 2 and Figure 4, both the overall and classification results showed obvious skewed distribution, with the mean larger than the median. The data with low spatial equity accounted for a higher proportion and had more concentrated distribution. Parks, squares, fitness centers and stadiums averaged 14.8, 20.2, 23.4 and 27.2, respectively. The average, median and high values of parks are relatively poor, but the standard deviation is the lowest, the overall data distribution is the most concentrated, and the internal gap is smaller than other types of sports space. The results of the square category showed obvious differences, with 50% data values lower than 7.9. The internal relative gap was the largest among all types, and the spatial distribution of sports was the most inequitable. The results of fitness center and stadium categories are similar, and the overall data structure is superior to the first two types of sports space. At the same time, the overall performance of the stadium category is slightly better than that of the fitness center category, reflected in larger mean and median. After the integration of the results of the 4 types, although there is still a relatively unequal situation, that is, 25% of residential buildings and the people in them enjoy a high level of sports space, the overall data structure has been improved, the relative gap of spatial fairness in urban areas has been narrowed to a certain extent, and the distribution of data has become more balanced.

### 3.3. Correlation Analysis between Walking and Public Transportation

In this study, sports space equity in walking and bus modes was discussed. As 2 independent variables, they respectively assessed the equity of people to sports space within a short distance (15-min walking) and a long distance (30-min bus travel), and the superposition results also represented the comprehensive situation of space equity. However, the correlation between them as independent variables has rarely been a part of previous studies. We conducted correlation analysis of the equity results in walking and public transportation modes according to classification of sports space and discussed the correlation level of the two traffic modes in central urban areas.

**Figure 5:**
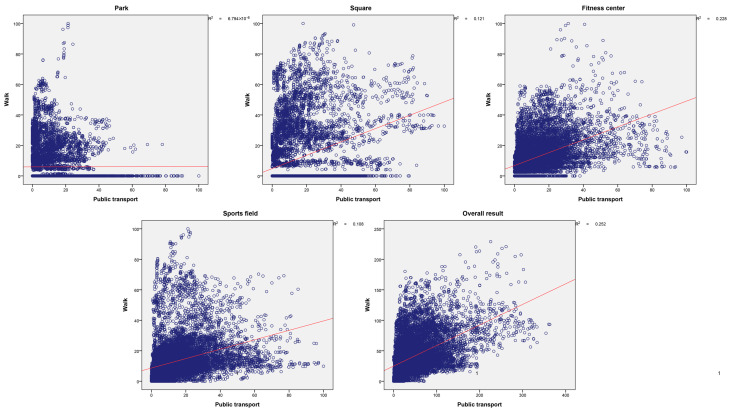
Scatter plot of spatial equity in walking and bus modes. The red line represents the linear relationship fitted by the scatter data.

As shown in Figure 5, the correlation results in different categories are different. Apart from parks, all of the other categories and the overall results pass the significance test (*p* < 0.01), indicating that the spatial equity of the people in the two modes is also closely related. The $r^{2}$ of square, fitness center, sports field and overall results were 0.121, 0.228, 0.108 and 0.252, respectively, showing significant correlation between the 2 modes. In other words, the equity of people having access to the sports space is similar in different traffic modes. When the equity value in the walking mode is low, the equity value in the bus travel mode is generally not high. The results of spaces such as parks did not pass the significance test, and the $r^{2}$ value was very low, indicating that there was no linear correlation between the influences of the two traffic modes on spatial equity. However, the $r^{2}$ value after overall superposition is the largest, indicating that when comprehensively considering multi-type sports space, the results of spatial equity calculated by the two traffic modes are similar. In other words, the spatial configuration of sports space, urban road network, traffic stations and other elements is actually inequitable. If people only reach the sports space by walking and bus, the status quo of inequity cannot be changed.

## 4. Discussion

The main contribution of this study is: based on previous studies, the multi-dimensional information is integrated into the classic model, including residential buildings, urban road network, transportation mode, multi-sports space, people’s preferences, and the impact of distance attenuation to obtain a more integrated and comprehensive application model. On the data level: POI data provide multiple spatial information of urban sports, which meets people’s different needs. Multivariate spatial analysis smooths the large internal gap that is easy to occur in a single type. The calculation results based on residential buildings are more precise and accurate, close to human scale and related to daily life. Dianping data is people’s intuitive assessment of sports space, quantifying the comprehensive quality of and people’s preference for the sports space. At the level of logical method: according to the concept of “life circle”, the equity standard of space equity is proposed to avoid different research scales caused by simple distance. Compared with other distance measurement methods, the network distance calculated on the basis of the GIS network analysis module is more realistic. The addition of distance attenuation function can more scientifically represent the change in spatial equity with the change in distance. Walking and public transportation are travel modes that can be adopted by all residents, which makes up for the shortcomings of single mode in previous studies.

**Figure 6:**
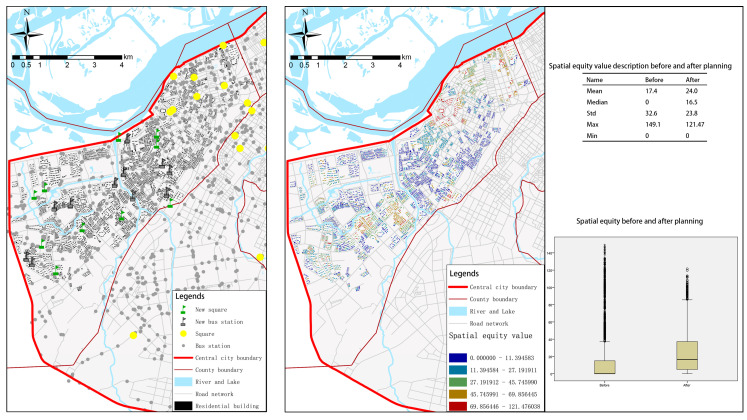
Comparison of the results before and after the spatial planning intervention of sports squares. Asterisks and circles represent outliers.

In this study, multi-dimensional visualization results were obtained to objectively show the status quo of sports space equity in central urban areas, which could support scientific planning intervention and decision-making. In the results of this study, squares have the biggest internal gap and the most inequitable overall situation among all of the types. Taking Daoli District as an example, we simply optimized the layout of facilities in combination with the concepts of the existing urban planning and life circle, including transforming existing vacant land, wasteland, garbage dump and other land with low utilization rate into squares and making up for areas with weak public transportation network. The results are shown in Figure 6. After spatial layout adjustment, the mean and median of spatial equity of squares increased significantly, the standard deviation became smaller, the overall distribution change was smoother, and the low value points also decreased significantly. It can be seen that targeted planning intervention can have a direct impact, and the spatial equity of the region has been positively optimized. However, we tend to overlook the problem and think that spatial equity is simply a homogeneous distribution of facilities or services, which can only remain in the ideal planning blueprint. According to previous studies, we need to consider the sports space with different functions and of different types [8,18,31], and different groups of people choose different sports space [1]. The starting point of spatial equity is to take people’s demand as orientation. We have obtained the status quo of spatial equity in the central urban area, which needs to be improved. However, before that, we need to understand the actual demand, for example, through the questionnaire of people’s demand, so as to make the most scientific and reasonable spatial planning.

There are still some limitations in this study. First, the urban road network is still relatively simple, and more attributes have not been added to it, such as driving speeds on different roads, waiting at junctions and traffic conditions. The assessment of bus travel mode is relatively simple and rough, which is a general estimate of people’s travel time. Second, this study only considered walking and public transportation. Adding more travel modes may narrow or increase the gap of spatial equity but will also be closer to reality. Thirdly, this study only calculated the public sports space open to the vast majority of people in urban areas but did not include some public facilities and services only for part of the population, such as sports venues in schools, residential areas, and government office areas, which will also affect the equity.

## 5. Conclusions

Taking Harbin as an example, this study presents the equity of public sports space in urban area based on the measurement method of multi-source urban data. The results show that there are significant inequities in the central urban area, the areas with high degree of equity are concentrated in the downtown and the junction of the three districts, and the emerging areas in the south of the city have the lowest degree of fairness. There are differences in the calculation results of different sports spaces. For example, the gap between park sports spaces is large and the overall situation is not ideal, indicating that there may be some problems in the previous park planning. Except for parks, the results of other types of sports Spaces were significantly correlated with the results of walking and bus modes, and the overall correlation degree was the highest. In other words, the areas with low walking equity degree also had low bus equity degree. Therefore, the planning of infrastructure should be considered in many aspects and promoted together to promote the equity of public sports space. In general, residents in urban centers tend to enjoy superior public sports space resources, while those in urban boundaries, the south and emerging areas do not. Therefore, the overall pattern should be optimized according to the distribution of key areas and specific types of sports space. In planning practice, it should also be weighed based on the needs of the population, so as to maximize the resource utilization of public sports space and meet the needs of sustainable development.

## Figures and Tables

**Table 1:** Statistical information and evaluation criteria of influencing factors of sports space.

Sports Space Classification	Amount	Service Properties	Service Capability Assessment		Attractiveness Assessment	
			Index	Interval	Index	Interval
Park	42	Free	Area	(1–5)	Dianping	(1–5)
Square	34	Free	Area	(1–5)	Dianping	(1–5)
Sports field	406	Paid/free	Grade	(1–5)	Dianping	(1–5)
Fitness center	377	Paid/free	Grade	(1–5)	Dianping	(1–5)

**Table 2:** Description of different types of sports space and comprehensive results.

Name	Mean	Median	Std	Max
Park	14.8	9.3	15.8	121.5
Square	20.2	7.8	27.0	160.5
Fitness center	23.4	17.7	21.6	140.3
Sports field	27.2	22.0	23.2	146.2
Overall result	21.4	17.6	17.8	126.0

## Data Availability

The datasets used and/or analysed during the current study are available from the corresponding author on reasonable request.
